# 3D Printed Paper-Based Microfluidic Analytical Devices

**DOI:** 10.3390/mi7070108

**Published:** 2016-06-28

**Authors:** Yong He, Qing Gao, Wen-Bin Wu, Jing Nie, Jian-Zhong Fu

**Affiliations:** 1 State Key Laboratory of Fluid Power and Mechatronic Systems, College of Mechanical Engineering, Zhejiang University, Hangzhou 310027, China; 11225005@zju.edu.cn (Q.G.); 3090102418@zju.edu.cn (W.-B.W.); 11525012@zju.edu.cn (J.N.); 2 Key Laboratory of 3D Printing Process and Equipment of Zhejiang Province, College of Mechanical Engineering, Zhejiang University, Hangzhou 310027, China; 3 State Key Laboratory for Manufacturing Systems Engineering, Xi’an Jiaotong University, Xi’an 710054, China

**Keywords:** 3D printing, paper-based microfluidic analytical devices (μPADs), flow speed programming

## Abstract

As a pump-free and lightweight analytical tool, paper-based microfluidic analytical devices (μPADs) attract more and more interest. If the flow speed of μPAD can be programmed, the analytical sequences could be designed and they will be more popular. This reports presents a novel μPAD, driven by the capillary force of cellulose powder, printed by a desktop three-dimensional (3D) printer, which has some promising features, such as easy fabrication and programmable flow speed. First, a suitable size-scale substrate with open microchannels on its surface is printed. Next, the surface of the substrate is covered with a thin layer of polydimethylsiloxane (PDMS) to seal the micro gap caused by 3D printing. Then, the microchannels are filled with a mixture of cellulose powder and deionized water in an appropriate proportion. After drying in an oven at 60 °C for 30 min, it is ready for use. As the different channel depths can be easily printed, which can be used to achieve the programmable capillary flow speed of cellulose powder in the microchannels. A series of microfluidic analytical experiments, including quantitative analysis of nitrite ion and fabrication of T-sensor were used to demonstrate its capability. As the desktop 3D printer (D3DP) is very cheap and accessible, this device can be rapidly printed at the test field with a low cost and has a promising potential in the point-of-care (POC) system or as a lightweight platform for analytical chemistry.

## 1. Introduction

As a lightweight analytical tool, paper-based microfluidic analytical devices (μPADs) have attracted increased interest and attention [1], which has led to their rapid development since Whitesides et al. [2] introduced the μPADs concept in 2007. Compared to conventional microfluidic chips made of glass and polymer substrates, μPADs have some unique advantages including, low cost, ease of fabricate, strong capillary action and good biological compatibility for applications in point-of-care (POC) diagnosis, food quality and environmental monitoring [3,4]. Many studies have explored fabrication techniques for μPADs including wax printing [5,6,7], inkjet printing [8,9], ink stamping [10,11], dynamic mask photo curing [12], paper cutting [13] and ink permanent markers [14] etc.

It is an ideal substrate for controlling flow and for confining liquids to specified areas without external pump on the paper. However, μPADs also suffer limited control over fluid transport, especially regarding the rate and direction of flow due to the physical properties inherent with porous substrates [3]. If the flow speed of μPADs can be programmed, the analytical sequences could be designed and they can be more easily used for performing multistep tasks or handling complex chemical matrixes. Several groups reported their progress on how to program liquid on μPADs, such as adjusting channel width [15,16,17,18] or carving microgrooves into the flow path to change the flow speed [19], adding button [20] or soluble fluidic barriers [21,22,23,24] as valves. Recently, some novel methods were also developed including using two-ply channels for faster wicking [25] and changing the permeability of channels to adjusting the flow speed [26].

Three-dimensional (3D) printing, also called additive manufacturing (AM) or rapid prototyping (RP), a relatively new approach for the fabrication of prototype 3D structures, has several advantages, such as the capability to print structures of complex shape, the combination of different materials, and less waste, low cost, and rapid production. Such advantages have made this rising technology a promising alternative way of fabricating microfluidic devices. Thus far, some methods and results have been reported about printing microfluidic chips [27,28,29,30,31,32,33,34,35,36,37,38,39]. Due to expiration of the key patents of fused deposition modeling (FDM) 3D printers and all of detailed technologies are open sourced, FDM printers are very popular and low-cost. It is also called the desktop 3D printer (D3DP) or personal 3D printer, as it may be accessible as easily as the personal computer [40].

Cellulose powder (α-Cellulose), a polysaccharide composed of long chains of β linked d-glucose units, is an organic compound with the formula (C_6_H_10_O_5_)n and the size of 74–125 µm. If cellulose powder is filled in a channel, it can transport liquid with capillary effect as the same as paper in our experiments. So in this report, we make an adequate use of the capillarity of the cellulose powder to fabricate a novel μPAD by adding the cellulose powder into the open microchannels on the 3D printed plastic substrate.

As the polymer substrate can be printed by the desktop 3D printer (D3DP), printing process is very convenient and its fabrication cost is very low. Additionally, since the D3DP may become as popular as the personal computer [40], this kind of microfluidic device can be rapidly manufactured in a common office, which is very suitable for the POC system. As this analytical device has the same working principle as the μPAD, and they can be directly 3D printed, we called this device as a μ3DPAD.

In this report, we demonstrated the flow speed of μ3DPADs can be programmed by adjusting the channel depth. Also, some analytical applications were also used to prove that μ3DPADs can be a good supplement of μPADs.

## 2. Materials and Methods

### 2.1. Materials

In this study, we used a D3DP, D-Force 400 (Trianglelab Co., Ltd., Qingdao, China), a typical FDM 3D printer, to fabricate the substrate with open channels. Polylactide (PLA) filament (PLA 1.75, Alkht Co., Ltd., Beijing, China) was used as the printing material. Polydimethylsiloxane (PDMS) (Sylgard 184, Dow Corning, Auburn, MI, USA) was spread over the channels to form a thin coated layer. Then the printed substrate with designed open channels, filled with mixture of cellulose powder (Sigma-Aldrich, Shanghai, China) and deionized water (Qianjing Environmental Technology Co., Ltd., Dongguan, China), was dried in an oven (DHG-9030A, Suoyu Equipment Co., Ltd., Shanghai, China). The solution of cellulose powder and deionized water was mixed on a magnetic stirrer (84-1A, Meiyingpu Equipment Co., Ltd., Shanghai, China) to ensure the uniform distribution of the cellulose powder. A standard solution of NaNO_2_ (GBW(E)080223, Beijing Aikeyingchuang Biotechnology Co., Ltd., Beijing, China) and an indicator solution for NO_2_^−^ were used to test the applicability of fabricated device.

### 2.2. Printing the μ3DPAD

**Figure 1:**
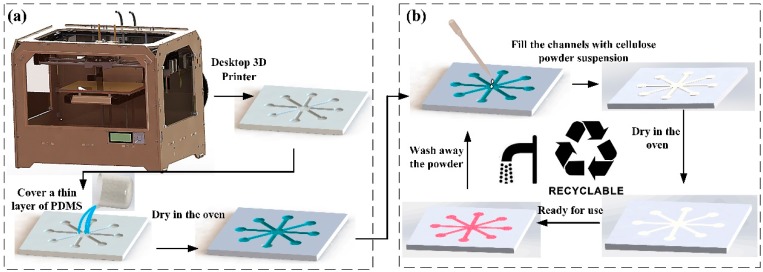
Fabrication process of microfluidic analytical device: (**a**) Substrate fabrication process; (**b**) Recyclable fabrication process of the hydrophilic cellulose powder channels.

The fabrication process of the μ3DPAD is shown in Figure 1. The whole process can be divided into two steps: the first step consists of printing the polymer substrate and the second step is the fabrication of the recyclable hydrophilic channels. Explicitly, in the first step, a 3D model of the microfluidic analytical device was designed using a 3D modeling software (e.g., SolidWorks, Unigraphics NX). The 3D model was then transmitted to the 3D printing software using the STL file format. After the substrate was printed by the D3DP, the surface of substrate was covered with PDMS to avoid permeation into the substrate by the following sample. After standing for 2 min, the PDMS totally penetrates into the flaws of the substrate. Subsequently, the excess PDMS was wiped off and the coated substrate was dried in an oven at 60 °C for 1 h to form a sealed, thin, hydrophobic layer. In the second step, the hollow channels on the substrate were filled with the mixture of cellulose powder and deionized water. The beaker with the mixture was placed on a magnetic stirrer and the rotating stirring bar helped the cellulose powder maintain in the deionized water. Because of the shrinkage effect, the cellulose powder mixture in the channels should be a bit higher than the depth of the channels. The substrate filled with the cellulose powder mixture was then dried in an oven at 60 °C for 30 min. When the fabricated device was taken out from the oven, it was ready for use.

## 3. Results and Discussions

### 3.1. Formation of the Hydrophilic Channels on the μ3DPADs

**Figure 2:**
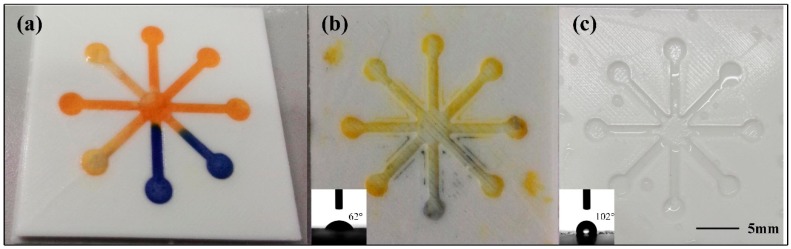
Comparison between the PDMS coated substrate and uncoated substrate: (**a**) PH test on the fabricated microfluidic device; (**b**) PDMS uncoated substrate washed by water after PH test (inset: water contact angle image of PDMS uncoated substrate); (**c**) PDMS coated substrate washed by water after PH test (inset: water contact angle image of PDMS coated substrate).

In the present work, the hydrophilic channels were formed on the hollow patterns of a hydrophobic substrate. The substrate was printed with a D3DP with PLA filament. Since we were aware that the D3DP has a big drawback in that the products cannot achieve a high surface quality compared to traditional injection molding technology. In addition, the PLA material does not possess a good hydrophobic property. Therefore, for an accurate experimental result, the patterned side of the printed substrate was designed to be covered with a layer of PDMS to improve its surface quality and hydrophobic property. To demonstrate the importance of the PDMS coating, we performed a comparison between the fabricated devices with coated and uncoated substrate, as shown in Figure 2. The measurement of PH was carried out on the fabricated microfluidic devices with coated and uncoated substrate. The two different types of microfluidic devices were washed with water and a brush. However, as shown in Figure 2b, the colored cellulose powder penetrated into the small gaps on the uncoated substrate surface and could not be easily washed off. On the other hand, the PDMS-coated substrate could be easily cleaned with water washing, as shown in Figure 2c.

**Figure 3:**
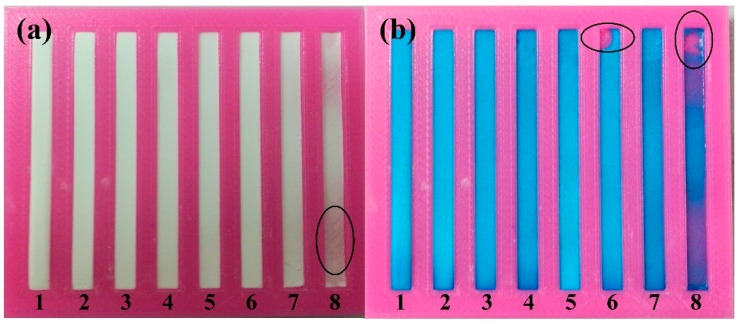
Cellulose powder channels fabricated with different proportion: (**a**) Comparison between the fabricated channels; (**b**) Blue dye was dropped to test the channels’ quality.

When filling the open channels with cellulose powder, we adapted a fabrication method similar to casting by transforming the state of the cellulose powder from solid into a turbid liquid. To investigate the effect of the proportion of cellulose powder and deionized water on the quality of the channels, eight straight cellulose powder channels were fabricated with different mixtures of cellulose powder and deionized water. The mass ratio of deionized water to cellulose powder, from channels 1 to 8, was 3:1, 4:1, 6:1, 8:1, 10:1, 12:1, 14:1, 16:1, respectively. We set the smallest ratio as 3:1 because the mixture could not flow freely in the channel with less deionized water, which may lead to failure in the fabrication of a uniform channel. The eight channels on the substrate were all 4 mm wide and 1 mm depth. The cellulose powder channels became thinner and thinner with increasing ratio, which caused the flaw indicated in channel 8, as shown in Figure 3. To fully evaluate the channels’ quality, blue dye was delivered into the eight channels, which caused the flaws indicated in channels 6 and 8. Thus, we recommend that the deionized water and cellulose powder ratio is between 3:1 and 10:1.

**Figure 4:**
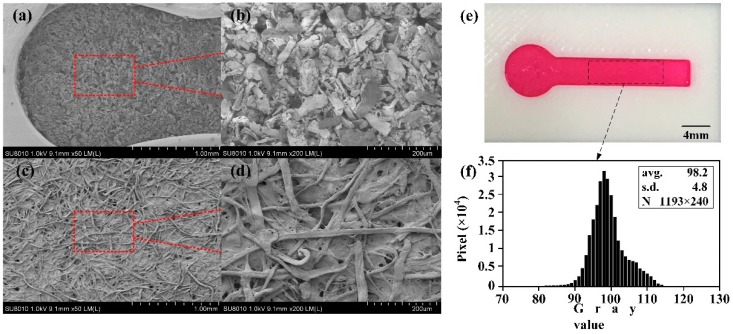
Scanning electron microscopy of cellulose powder in fabricated device and Whatman No. 1: (**a**) Microstructure of cellulose powder under microscope (50×); (**b**) Microstructure of cellulose powder under microscope (200×); (**c**) Microstructure of chromatography paper Whatman No. 1 under microscope (50×); (**d**) Microstructure of chromatography paper Whatman No. 1 under microscope (200×); (**e**) A dying test on a μ3DPAD with a channel of 4 mm width; (**f**) Gray value distribution of the dye in the channel.

The capillary force is the driven force of μPAD and μ3DPAD. So the microstructure of the cellulose powder in the channels and the filter paper, Whatman No. 1 which is always used to fabricate the μPAD, are investigated with comparison, as shown in Figure 4a–d. Compared to the Whatman No. 1, the surface morphology of the cellulose powder is more ordered, which is benefit to keep a steady flow and a uniformity of solution dispersion at the microscale. Figure 4e shows a dying test on a μ3DPAD with a channel of 4 mm width. The gray value was analyzed by Matlab (R2014a, The MathWorks, Inc., Natick, MA, US) of 1193 × 240 pixels. As shown in Figure 4f, the coefficient of variance (CV = SD/avg.) of the gray value is computed to be 4.9%, which means that the dye dispersion in the channel is uniform.

### 3.2. Printing Resolution of the μ3DPADs

**Figure 5:**
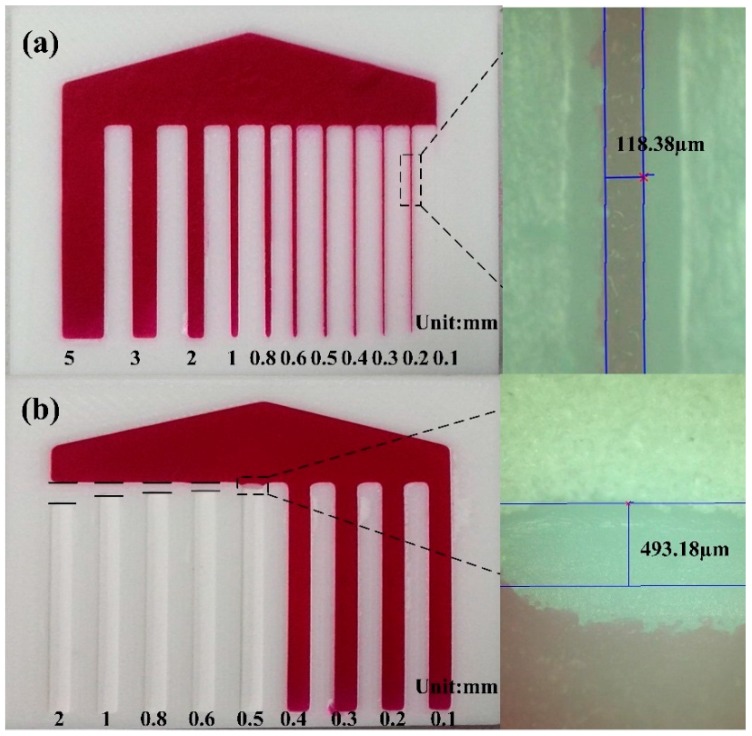
Resolution of μ3DPADs: (**a**) The resolution of the hydrophilic channels and the channel’s image under the microscope (100×); (**b**) The resolution of the hydrophobic barriers and the barrier's image under the microscope (100×).

Although the D3DP is inexpensive and easily accessible, the printing resolution is its biggest drawback. The question is: is D3DP adequate for printing the μ3DPAD? Thus, we performed various experiments to investigate the resolution of the μ3DPADs and compare it with the μPADs. The minimum size of hydrophilic channels was 118 ± 17 µm, while that for the between-channel hydrophobic barrier was 493 ± 22 µm, as shown in Figure 5. Accordingly, as the typical resolution of hydrophilic channels and hydrophobic barrier of the μPAD is about 500 µm [1], the D3DP can provide an adequate resolution for the fabrication of μ3DPADs.

### 3.3. Speed Programing by Adjusting Channel Depth

With the help of 3D printing, it is easy to print a 3D sharp channel, so adjusting the channel depth to program the flow speed becomes possible. Two factors determine the flow time of liquid in a channel, namely channel width and channel depth. Adjusting the channel width to acquire different flow speeds on μPADs had been investigated by some studies [15,16,17,18]. Here we only describe a research conducted to examine the relationship between channel depth and solution flow rate under the driving of capillary force.

**Figure 6:**
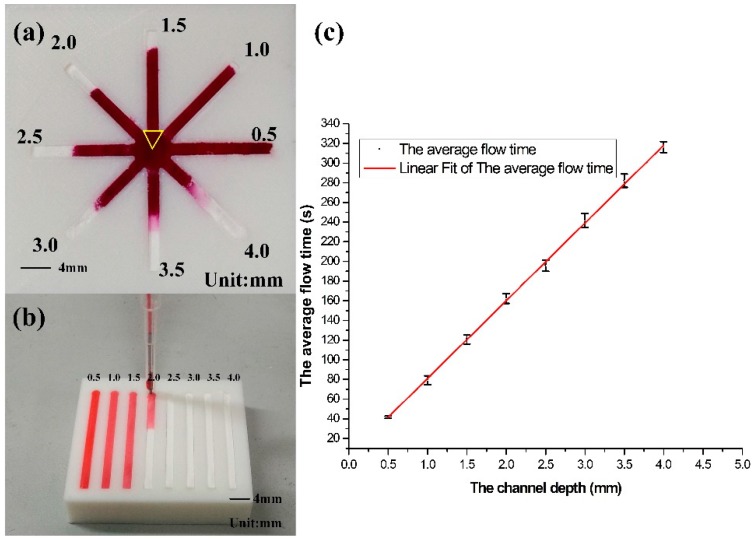
Relationship between channel depth and flow time: (**a**) The flow trend of red dye in 8 channels with a gradient depth; (**b**) Quantitative analysis on the relationship between channel depth and flow time; (**c**) The linear relationship of speed and the depth.

The results revealed that when red dye was delivered into the center of a μ3DPAD, the flow trend corresponded with depth of the channel, as shown in Figure 6a. Additionally, a special device was fabricated to investigate the quantitative relationship between channel depth and solution flow rate. The eight channels on the device are all 2 mm wide and 30 mm long and have a depth gradient from 0.5 to 4 mm, as shown in Figure 6b, The red dye was dropped by a syringe pump with a flow rate of 1 mL/h into the inlet of each channel with a depth gradient from 0.5 to 4 mm. Four groups of data on the relationship between channel depth and solution flow time are listed in Table 1. A linear fit of the average flow time was built according to these data, as shown in Figure 6c, the flow speed exhibits a linear relationship with the channel depth. Thus, it is very easy and convenient to control the flow rate by controlling the channel depth in order to program the flow rate of the microfluidic analytical device.

**Figure 7:**
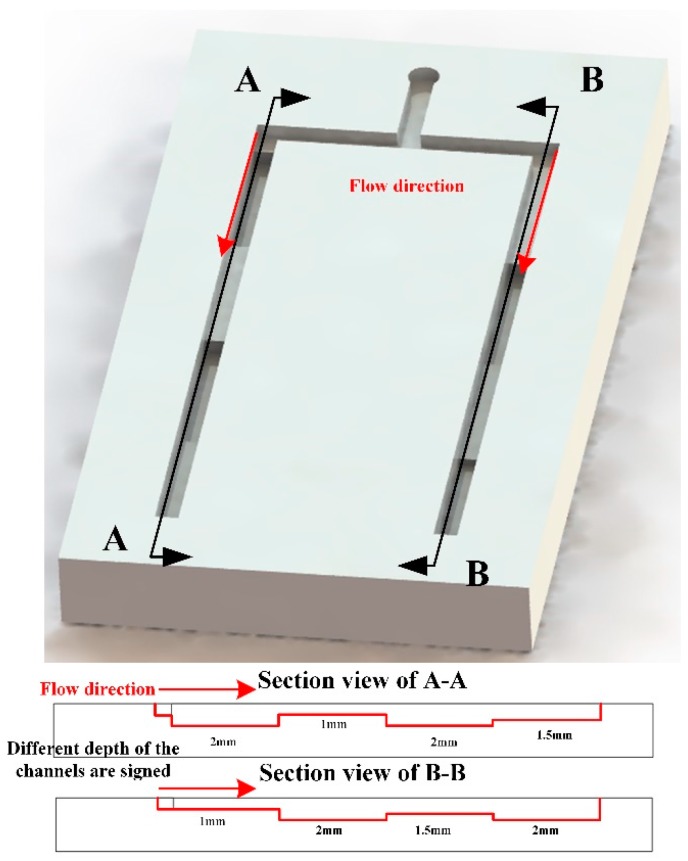
Flow speed control in 3D channels. The left channel and the right channel all have four segments with the different depth and the same segment length.

To better demonstrate the flow speed can be programmed, we printed a μ3DPAD with variable channel depth at different places, as shown in Figure 7. The left channel and the right channel all have four segments of the different depths, but the same length, as shown in Table 2. As the flow speed has a linear relationship with the channel depth, so the flow speed of two channels will be different, but it should reach the end at the same time theoretically. From the Table 2, we can find that the experiments fit the theoretical prediction well. In the other word, the flow speed of μ3DPAD can be well designed according to the analytical requirements.

### 3.4. Encapsulation of the μ3DPADs

**Figure 8:**
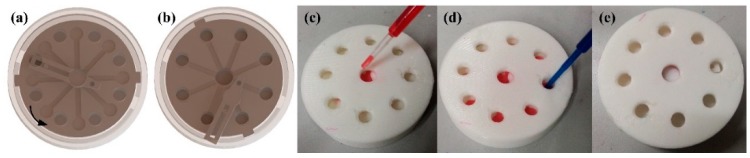
The encapsulation of the microfluidic analytical device: (**a**) Model graph of the device in closed state; (**b**) Model graph of the device in open state; (**c**) Dropping indicating solution in physical model in open state; (**d**) Dropping test solution in physical model in open state; (**e**) Physical model graph of the device in closed state.

As an open channel microfluidic analytical device, unprotected channels are potential sources of sample contamination, particularly when it is directly exposed to the air. As the μ3DPAD is fabricated by D3DP, it is very easy to print an integrated μ3DPAD with a protecting mask. We designed a set of NO_2_^−^ test encapsulation equipment to demonstrate that encapsulation is very easily performed by the D3DP. Figure 8 shows how the equipment works. The analytical part was set in an encapsulation equipment with nice holes for observing and installing. The analytical parts can rotate in the encapsulation equipment to switch its state from closed to open by a spatial linkage. The detection zones are located between the holes and the central zone is covered by the spatial linkage, as can be seen in Figure 8a. Swinging the analytical device anticlockwise, the detection zones are under the holes and the spatial linkage uncovers the central zone. In Figure 8c,d, the red dye represents the indicator solution and the blue dye represents the test solution. As we can see from this example, with 3D printing technology, the encapsulation of the μ3DPADs can be easily realized.

### 3.5. Fabrication of T-Sensor

**Figure 9:**
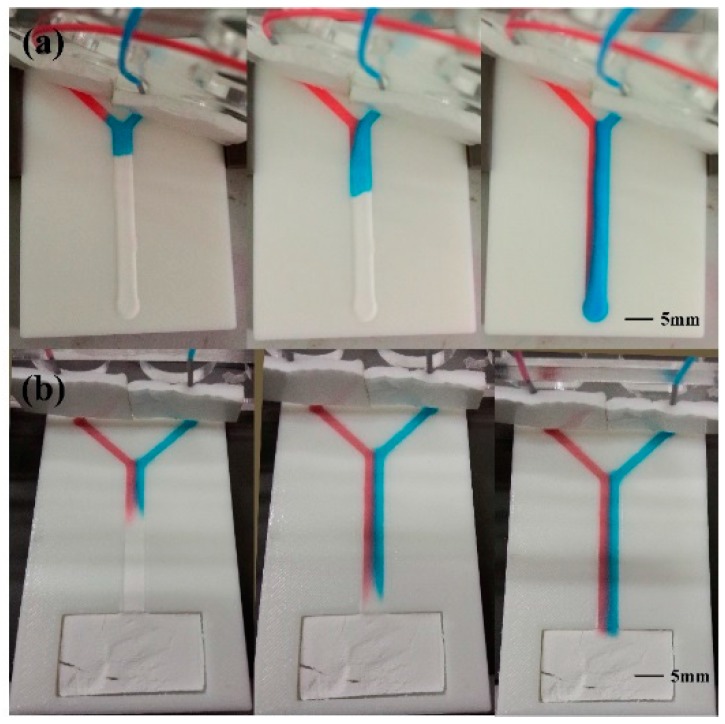
Time-lapse image of two different dyes diffusing in the fabricated Y device: (**a**) Microfluidic Y device with two different fluid path lengths; (**b**) Microfluidic Y device with the same path length.

Traditional microfluidic analytical devices that exploit transverse diffusion across adjacent flow stream are Y (or T) structures which combine multiple inlet streams into a common channel. These geometries have been developed to study inter diffusion of one or multiple species, to evaluate transport processes, and to detect analytes ranging from pH to immunoassay targets [41]. To achieve such applications with traditional microfluidic analytical devices requires at least two extremely stable pumps to inject two different solution from two inlets. Osborn, et al. developed an alternative pumpless microfluidic Y device with paper [16]. We fabricated two kinds of Y devices to verify the flow control capacity of the μ3DPADs. For simplicity, here we used an injection pump with two connecting tubes to drop two different dyes at the same rate, to the two inlets. Actually, as the device was capillarity driven, the injection pump was not used to drive the liquid, it was just used to provide the dye, which means other devices that can provide two similar flows such as the source pad were also effective. The width of the inlets and the common channels was 2 and 4 mm, respectively, while the depth of all the channels was 0.5 mm. Additionally, the length from the inlets to the junction were 15.5 and 4.5 mm, respectively, as shown in Figure 9a. The stream width of the red and blue dyes were 0.9 and 3.1 mm, respectively. Since the rate of the two flows was the same, the width of the stream was determined by the length of the inlet arms. As shown in Figure 9b, in addition, the length from the inlets to the junction were both 15.5 mm. The stream width of the red and blue dyes were both 2 mm. According to the Darcy’s law, the ratio of the red dye inlet to blue dye inlet arm length is the same as the ratio of the blue dye to red dye stream width. The result obtained here mostly matched the Darcy’s law, which proves that the fabricated cellulose powder channels had a uniform distribution and were capable of achieving precise flow experiments.

### 3.6. Assay of Nitrite Concentration

To demonstrate the practicability of the μ3DPAD in chemical detection, a fabricated device with the new method was evaluated using quantitative analysis as a case study. This μ3DPAD had eight detection zones and a central zone. When all detection zones were filled with the NO_2_^−^ indicator solution, 7 of them were pipetted with the standard nitrite solution and the last one was pipetted with the sample solution of a known concentration.

We used a simple and convenient method to perform the NO_2_^−^ assay with the μ3DPAD that has eight detection zones. First, 40 µL of the colorless NO_2_^−^ indicator solution was pipetted into the central zone, the bigger circle in the middle. When the indicator solution completely penetrated the detection zones, 10 µL portions of the standard solutions and the prepared sample solution were individually pipetted into the detection zones. The analytical device was photographed after the color reaction had completely developed. Then the photo was converted to grayscale with Adobe Photoshop CS5 (Adobe Systems Inc., San Jose, CA, USA). The curve depicting the relationship of the measured gray intensities and nitrite concentration was constructed to calculate the concentration of the sample solution. The detection result was compared to the real concentration to verify the efficacy of the fabricated analytical device.

**Figure 10:**
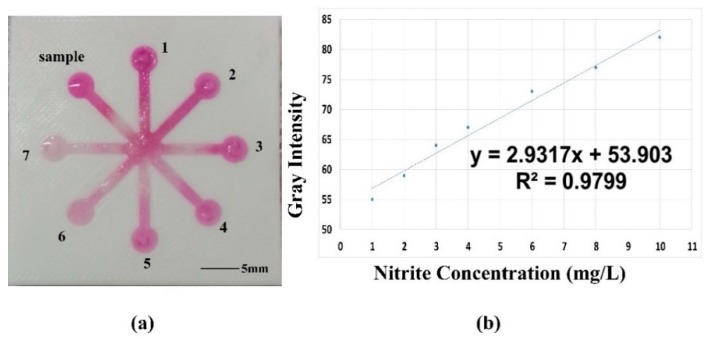
Colorimetric assay of nitrite via color-reaction by using microfluidic analytical device: (**a**) Image of the testing microfluidic analytical device; (**b**) Curve for nitrite ion.

The nitrite concentration of each sample is listed in Table 3. The results revealed that the 1–7 detection zones reflected the color reaction, as shown in Figure 10a. The acquired image was turned into grayscale and the data was collected, and are listed in Table 3. A curve depicting the relationship between the nitrite concentration and the corresponding gray intensity was obtained by fitting with the least squares method. The curve was constructed and the regression equation obtained is *y* = 2.9317*x* + 53.903 with an *R*^2^ of 0.9799, which shows the line was fitted well, as shown in Figure 10b. According to this equation, the gray intensity is 74 and its corresponding testing result is 7.44 mg/L. Such results indicate that, compared to the real concentration of 7 mg/L, the designed microfluidic analytical device demonstrates an adequate capability for quantitative analysis.

## 4. Conclusions

In this study, a novel microfluidic analytical device driven by capillary force, namely the μ3DPAD, is presented as a good supplement for the μPAD in many applications, with the improvements of flow speed control, easy fabrication, and easy encapsulation. The investigation of the relationship between channel depth and flow time, performed to achieve flow rate programming, indicated that it is easy to control the flow speed in a certain range with this method.

Although the μ3DPADs shows some promising features such as easy fabrication, programmable flow, some limits about the cellulose powder should be addressed. After filling cellulose powder liquid in the open channels, shrinkage could cause some cracks in large filling areas after liquid dried. If the open channels’ depth is over 2 mm, we suggested to fill cellulose powder with two times. If the cellulose powder or some other materials that have capillary effect could be fabricate in cream, then it can be smeared on the open channels and fabrication the μ3DPADs will be more convenient.

## Figures and Tables

**Table 1:** Dye flow time in channels with different depth.

Channel Depth (mm)	0.5	1.0	1.5	2.0	2.5	3.0	3.5	4.0
Flow time1 (s)	42.80	75.90	117.10	158.00	187.70	231.10	274.50	310.00
Flow time2 (s)	42.20	81.10	123.60	165.30	198.20	245.50	280.30	320.30
Flow time3 (s)	41.40	75.20	116.40	158.40	200.80	246.50	282.70	321.50
Flow time4 (s)	40.20	84.70	125.50	167.60	196.20	243.20	290.70	313.30
The average flow time (s)	41.65	79.23	120.65	162.33	195.73	241.58	282.05	316.28
Standard deviation	1.12	4.50	4.58	4.86	5.67	7.12	6.72	5.53

**Table 2:** Flow time of two channels.

Position	Depth of the Left Channel (mm)	Depth of the Right Channel (mm)	Flow Time in the Left Channel (s)	Flow Time in the Right Channel (s)
First step	2	1	68	46
Second step	1	2	98	99
Third step	2	1.5	155	140
Fourth step	1.5	2	189	190

**Table 3:** Nitrite concentration of samples 1–7 and their corresponding gray intensity.

Sample	1	2	3	4	5	6	7
Gray intensity	82	77	73	67	64	59	55
Nitrite concentration, mg/L	10	8	6	4	3	2	1
